# Simultaneous ^166^Ho/^99m^Tc dual-isotope SPECT with Monte Carlo-based downscatter correction for automatic liver dosimetry in radioembolization

**DOI:** 10.1186/s40658-020-0280-9

**Published:** 2020-03-04

**Authors:** R. van Rooij, A. J. A. T. Braat, H. W. A. M. de Jong, M. G. E. H. Lam

**Affiliations:** Department of Radiology and Nuclear Medicine, University Medical Center Utrecht, Heidelberglaan 100, 3584 CX Utrecht, The Netherlands

**Keywords:** Radioembolization, SIRT, Ho-166, Radiocolloid, Dosimetry

## Abstract

**Background:**

Intrahepatic dosimetry is paramount to optimize radioembolization treatment accuracy using radioactive holmium-166 microspheres (^166^Ho). This requires a practical protocol that combines quantitative imaging of microsphere distribution with automated and robust delineation of the volumes of interest. To this end, we propose a dual isotope single photon emission computed tomography (SPECT) protocol based on ^166^Ho therapeutic microspheres and technetium-99 m (^99m^Tc) stannous phytate, which accumulates in healthy liver tissue. This protocol may allow accurate and automatic estimation of tumor-absorbed dose and healthy liver-absorbed dose. The current study focuses on a Monte Carlo-based reconstruction framework that inherently corrects for scatter crosstalk between the ^166^Ho and ^99m^Tc imaging. To demonstrate the feasibility of the method, it is evaluated with realistic phantom experiments and patient data.

**Methods:**

The Utrecht Monte Carlo System (UMCS) was extended to include detailed modeling of crosstalk interactions between ^99m^Tc and ^166^Ho. First, ^99m^Tc images were reconstructed including energy window-based corrections for ^166^Ho downscatter. Next, ^99m^Tc downscatter in the 81-keV ^166^Ho window was Monte Carlo simulated to allow quantitative reconstruction of the ^166^Ho images. The accuracy of the ^99m^Tc-downscatter modeling was evaluated by comparing measurements with simulations. In addition, the ratio between ^99m^Tc and ^166^Ho yielding the best ^166^Ho dose estimates was established and the quantitative accuracy was reported.

**Results:**

Given the same level of activity, ^99m^Tc contributes twice as many counts to the 81-keV window than ^166^Ho, and four times as many counts to the 140-keV window, applying a ^166^Ho/^99m^Tc ratio of 5:1 yielded a high accuracy in both ^166^Ho and ^99m^Tc reconstruction. Phantom experiments revealed that the accuracy of quantitative ^166^Ho activity recovery was reduced by 10% due to the presence of ^99m^Tc. Twenty iterations (8 subsets) of the SPECT/CT reconstructions were considered feasible for clinical practice. Applicability of the proposed protocol was shown in a proof-of-concept case.

**Conclusion:**

A novel ^166^Ho/^99m^Tc dual-isotope protocol for automatic dosimetry compensates accurately for downscatter and allows for the addition of ^99m^Tc without compromising ^166^Ho SPECT image quality.

## Background

**Fig. 1:**
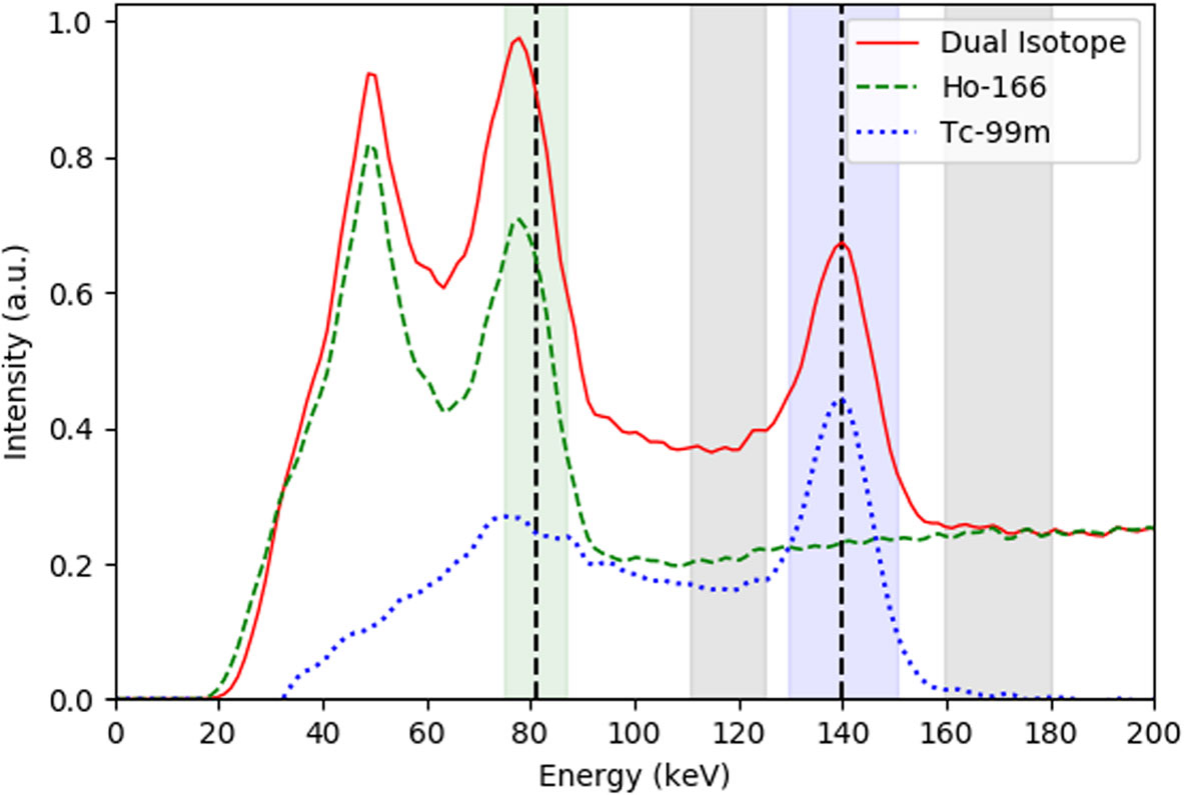
^166^Ho-only spectrum of a patient scan (dashed green line), dual-isotope spectrum of the same patient after administration of ^99m^Tc (solid red line), difference between the two spectra, representing ^99m^Tc only (dotted blue line). Recorded energy windows are shaded. Both isotopes contribute a significant amount of scatter to one another’s photopeak window

Radioembolization has rapidly developed over the past decade. Conventionally, the amount of injected activity is based either on the patients body surface area or on the target liver volume for the commercially available resin and glass yttrium-90-loaded microspheres, respectively (SirSpheres® from Sirtex Medical and Therasphere® from BTG International). These methods are applied under the assumption that microsphere distribution is homogenous in the treated volume. However, due to patient characteristics and especially the heterogeneity of the microsphere distribution, these methods are too simplistic to allow for reliable dosimetry. In recent years, more and more centers have adopted the partition model, defining a tumor and non-tumor compartment, and allowing more personalized activity calculation by comparison with minimal required tumor dose and maximum allowable healthy liver dose from literature. Although much more accurate, the downside of the partition model is the delineation of the compartments, which is usually done manually. This can be cumbersome and hampers clinical widespread adoption. An automatic protocol could solve this. For radioembolization treatments with holmium-166-loaded (^166^Ho) microspheres (Quiremspheres®, Quirem Medical), we propose a dual-isotope SPECT/CT protocol using pretreatment ^166^Ho scout dose as treatment simulation and technetium-99m stannous phytate (a radiocolloid) for healthy liver tissue delineation [1]. ^99m^Tc-stannous phytate only accumulates in Kupffer cells by phagocytosis of the stannous phytate particle. As Kupffer cells are absent in tumorous tissue, this radiopharmaceutical has been used for many decades for the detection of liver disease and liver malignancies. The main advantage of simultaneous SPECT acquisition of both the treatment simulation with ^166^Ho microspheres and healthy liver tissue segmentation with ^99m^Tc colloid is the absence of miss registration, due to patient-related factors. This manuscript will focus on the technical challenges concerning image acquisition and reconstruction with this dual-isotope protocol, mainly related to crosstalk of the two isotopes. Accurate quantitative reconstruction of ^166^Ho SPECT has been demonstrated in previous work by Elschot et al. [2], but the presence of ^99m^Tc during the acquisition causes a significant contamination in the ^166^Ho energy window. Vice versa, the ^99m^Tc photopeak window is contaminated due to downscatter from high energy ^166^Ho emissions. This crosstalk interaction is illustrated in Fig. 1, depicting an energy spectrum of both ^166^Ho and ^166^Ho + ^99m^Tc. The Utrecht Monte Carlo System (UMCS) was extended to be able to correct for these crosstalk interactions.

## Materials and methods

### Implementation of the photon modeling

In previous work, Elschot et al. demonstrated an iterative OSEM reconstruction method (Utrecht Monte Carlo System, UMCS) for quantitative ^166^Ho SPECT, which includes the Monte Carlo-based modeling of photon contributions from the full ^166^Ho energy spectrum, including bremsstrahlung [2]. In short, besides Compton and photo-electric effects in the patient, fast simulation of all collimator and crystal effects was accomplished by incorporating a look-up-table of point spread functions (PSF), which were generated with MCNP, a general purpose Monte Carlo radiation transport code [3]. For this work, this method was extended to include the effect of ^99m^Tc downscatter in the 81-keV photopeak window of ^166^Ho in a similar fashion. The PSF as detected in the 81-keV (15% width) energy window depends on the energy of the photons before the detection and the distance to the collimator. To represent this, multiple PSFs were simulated for the Siemens Symbia Medium Energy collimator at source-detector distances of 1 cm, 5 cm, 12 cm, 24 cm, and 40 cm; PSFs for intermediate distances are interpolated at runtime. In order to restrict computation time, PSFs for 8 specific energies are used to cover the full energy spectrum. Each photon is associated with a PSF, determined by its energy as illustrated in Table 1. These PSFs represent photons that have an initial energy outside the 81-keV window and require a collimator or detector interaction (e.g., partial energy deposition, lead x-ray emission) to generate a detection. In addition to the PSFs for these indirect detections, a separate PSF was generated to represent photons that are “directly” detected, i.e., photons that have an energy in the 81-keV window (e.g., after scattering in the patient). These events are weighted by the detection probability, determined by the energy of the photon relative to the energy window and the energy resolution of the gamma camera.

**Table 1:** UMCS photon energies and their associated PSF (denoted by the pre-simulated source energy) for detection in the 81-keV (15% width) energy window

Photon energy (keV)	Associated PSF source energy (keV)
60–88	81
88–106	95
106–129	118
129–154	140
154–226	170
226–462	300
462–992	713
992–2000	1379

### Image reconstruction and validation

Image reconstruction of a dual-isotope acquisition was performed in three consecutive steps, in which UMCS was used as the forward projection simulator for each step.

Firstly, ^99m^Tc reconstruction: crosstalk of ^166^Ho in the 140-keV ^99m^Tc photopeak window was corrected for during iterative reconstruction by addition of an upper scatter window (centered at 170 keV). A *k*-factor of 0.93 (or 0.96 including the window width ratio) was applied to account for the slightly decreased ^166^Ho scatter contribution around 140 keV (which can be appreciated from the small slope in the ^166^Ho spectrum in Fig. 1 between 140 keV and 170 keV). The *k*-factor was determined as the mean count ratio between the projection images of these energy windows in 65 patient SPECT acquisitions.

Secondly, ^99m^Tc downscatter: using the ^99m^Tc reconstruction, projection images of ^99m^Tc downscatter into the 81-keV ^166^Ho photopeak window were simulated.

Thirdly, ^166^Ho reconstruction: crosstalk from ^99m^Tc was corrected for by adding the simulated ^99m^Tc-downscatter projections as a scatter window during iterative reconstruction of ^166^Ho.

### Evaluation

The crosstalk simulation and performance of the image reconstructions were assessed by conducting and comparing phantom studies. A ^99m^Tc line source centered between two 40 × 40 × 10 cm^3^ slabs of polymethyl methacrylate (PMMA) scatter material and a 6.3-L cylindrical phantom, filled with 50 MBq ^99m^Tc, were scanned on a Siemens Symbia T16 SPECT/CT, recording the clinically used energy windows of both ^99m^Tc (140 keV, 15% width) and ^166^Ho (81 keV, 15% width), along with two scatter windows centered at 118 keV and 170 keV (widths 12%). The recorded projections of both phantoms were compared with simulated projections of a digital phantom (of equal shape and activity, based on the attenuation CT image of the setup) to assess quantitative accuracy of the ^99m^Tc-downscatter simulations (i.e., the extent to which ^99m^Tc contaminates the 81-keV ^166^Ho photopeak window). Projections of the ^99m^Tc line source were used to validate the shape of the PSFs corresponding to ^99m^Tc downscatter in the 81-keV energy window.

### Determination of the ^166^Ho–^99m^Tc activity ratio

**Fig. 2:**
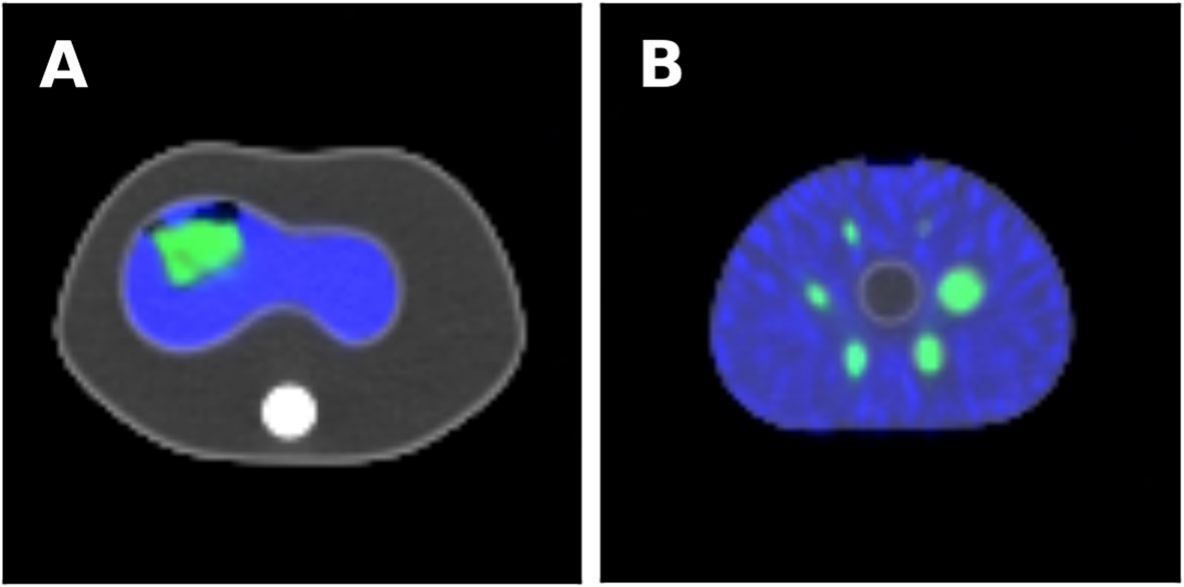
Dual-isotope SPECT reconstructions (^166^Ho in green, ^99m^Tc in blue) fused with the accompanying CT images (grayscale). **a** Anthropomorphic torso phantom with the 1200-ml liver compartment filled with a 34-kBq/ml ^99m^Tc solution. A 130-ml insert, filled with a 0.4-MBq/ml ^166^Ho solution, was placed inside of the liver compartment. The remaining volume of the phantom was filled with water. **b** NEMA image quality phantom, background filled with an 11-kBq/ml ^99m^Tc activity concentration. The spheres were filled with a 0.8-MBq/ml ^166^Ho solution

To define a practical balance between the amount of administered ^166^Ho and ^99m^Tc, an anthropomorphic torso phantom was measured (Fig. 2a). Within the 1200-ml water filled liver compartment of the phantom, a 130-ml insert was placed containing a 53-MBq ^166^Ho solution. Two scans were performed. In the first scan, the phantom contained ^166^Ho only, for the second scan, 35 MBq ^99m^Tc was added to the liver compartment. The data was reconstructed with our protocol and analyzed for quantitative accuracy for both the ^166^Ho only scan and the dual-isotope scan. A clinically acceptable ^166^Ho/^99m^Tc activity ratio was defined, based on the results of the latter phantom study and on a visual interpretation and consensus reading by two nuclear medicine physicians and a medical physicist.

### Quantitative assessment

The quantitative reconstruction accuracy of a^166^Ho activity distribution is dependent on the size of the distribution and is further influenced by the presence of ^99m^Tc. The National Electrical Manufacturers Association (NEMA) image quality (IQ) phantom (Fig. 2b) was used to determine the sphere size-based activity recovery for 3 different ^99m^Tc background concentrations. The 6 spheres in the NEMA IQ phantom (diameters 10, 13, 17, 22, 28, and 37 mm, volumes, 0.52, 1.15, 2.6, 5.6, 11.5, and 26.5 ml) were filled with a 0.8-MBq/ml ^166^Ho solution and 3 acquisitions were performed with varying ^99m^Tc activity concentrations in the background compartment (0, 6, and 11 kBq/ml). The phantom was partially filled with agar-agar to reduce the total volume of the background compartment (5.5 L instead of the conventional 9.7 L), in order to obtain a ratio between the total activity and the activity concentration that resembles a clinical situation more closely. Images of the ^166^Ho activity distribution were reconstructed using the dual-isotope reconstruction protocol described above. Volumes of interest (VOIs) were placed over the spheres, where the size of each VOI matched the size of the corresponding sphere in the phantom. The positioning of the VOIs was performed automatically (using a fit routine to optimize the mean activity within the VOIs). The mean ^166^Ho activity concentration within each VOI was divided by the known injected activity concentration to obtain the activity recovery coefficients. Because in clinical practice, the actual shape or volume of an activity deposition is not necessarily known beforehand, a VOI may be drawn wider around the hot spot to determine the total activity within the spot. To emulate this, additional to the VOIs that matched the sphere sizes, the diameters of the VOIs were increased by 20 mm. The increase of 20 mm was found to be sufficiently large to limit spill-out due to the partial volume effect, while not being so large as to overlap with neighboring spheres. Furthermore, activity recovery coefficients were computed for all spheres and for each iteration in the reconstruction (100 iterations using 8 subsets of 15 projections, 120 angles in total) to investigate the influence of the number of iterations on the quantitative accuracy of the reconstruction protocol.

### Proof of concept in clinical setting

If a patient is a candidate for radioembolization, first, a visceral angiography is performed to assess the arterial blood supply of the liver and tumors. During the same angiography, positioning of the microcatheter for a radioembolization treatment is determined by the interventional radiologist. To simulate the actual radioembolization treatment, a scout dose of 250 MBq ^166^Ho microspheres is administered in the pre-determined microcatheter positions. Subsequently, a SPECT/CT is acquired to assess treatment safety (i.e., excluding extrahepatic depositions of activity) and assess the intrahepatic distribution of the particles for treatment dosimetry. As part of a prospective clinical study (HEPAR PLUS), informed consent for the acquisition of the proposed dual-isotope SPECT/CT in a patient was obtained [4]. After a regular ^166^Ho scout dose procedure (with 250 MBq), a ^166^Ho-only SPECT/CT was acquired. Subsequently, 10 min after intravenous injection of 50 MBq ^99m^Tc-stannous phytate, a dual-isotope SPECT/CT was acquired. The imaging protocol of the dual-isotope SPECT/CT was based on the results of our phantom study.

## Results

**Fig. 3:**
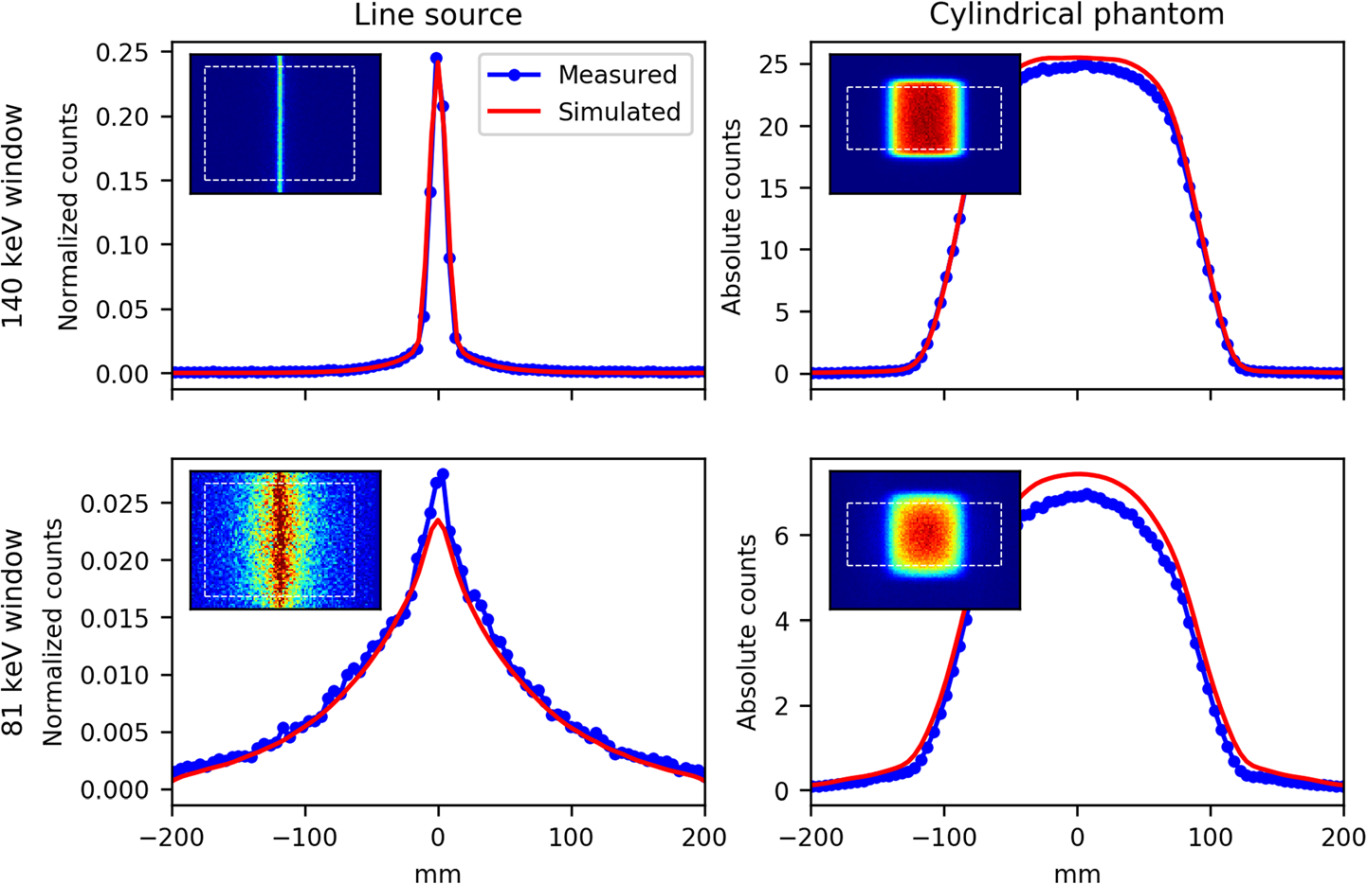
Line profiles of ^99m^Tc line source and ^99m^Tc cylindrical phantom, with the corresponding projections in the upper left corners. Both recorded in the 140-keV energy window (top row) and the 81-keV energy window (bottom row). The line profiles of the line source (left column) were obtained by summing along the length of the line source as indicated by the dashed box. Both profiles were scaled to normalize the summed intensity in the 140-keV window for the measurement and simulation independently. The line profiles of the cylindrical phantom (right column) were obtained by averaging the projection images over all angles (120 angles over a 360-degree rotation), and subsequently averaging along the length of the phantom indicated by the dashed box

Projection images of the ^99m^Tc line source in the 81 keV and 140-keV energy windows were simulated and compared with the measured projection images (Fig. 3, left column). Comparing the number of counts in the 81-keV window (C_81_) with the number of counts in the 140-keV window (C_140_), the simulation underestimated C_81_/C_140_ by 8% (0.59 simulated versus 0.64 measured).

Quantitative accuracy of the ^99m^Tc forward projections in the 140 keV and the 81-keV window was also assessed using the cylindrical phantom. A homogeneous activity distribution was imposed inside the digital phantom, matching the total activity as measured in a dose calibrator (Fig. 3, right column). The simulated projections overestimated the counts in the 140-keV energy window by 3.5%, and the counts in the 81-keV window by 10.7%. Since no cross-calibration was performed between the dose-calibrator and the scanner, the simulated projections were intrinsically quantitative (i.e., unscaled).

The projection images of the anthropomorphic phantom showed that, for an equal amount of activity, ^99m^Tc contributes approximately twice as many counts to the 81-keV window as ^166^Ho, and 4 times as many counts to the 140-keV window. These numbers strongly depend on the distribution of the isotopes and the geometry of the patient. Based on the results of the anthropomorphic phantom study and the consensus reading, a clinical activity ratio of 5:1 (250 MBq ^166^Ho:50 MBq ^99m^Tc) was chosen. Thus, counts contributing to the 81-keV window are mostly due to ^166^Ho (5:2), while at the same time the intensity in the 140-keV window is approximately balanced between ^166^Ho and ^99m^Tc (5:4). The ^166^Ho only reconstruction of the anthropomorphic phantom overestimated the activity by 7% compared with the calibrated injected activity. Addition of ^99m^Tc in the liver compartment further reduced the accuracy, overestimating the ^166^Ho activity by 14%.

**Fig. 4:**
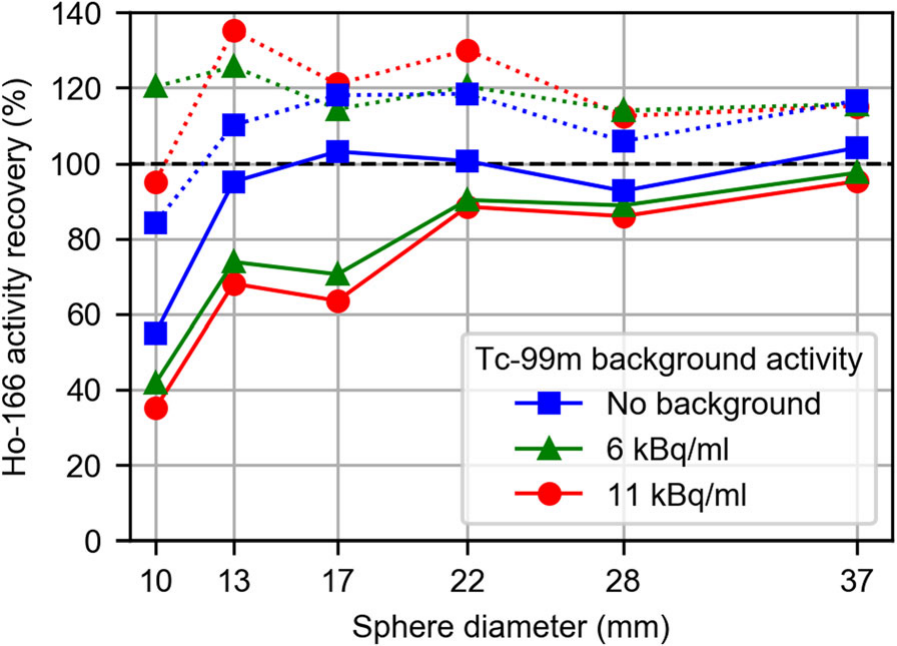
Recovered ^166^Ho activity as a percentage of the known injected activity for ^166^Ho filled spheres in the NEMA image quality phantom, after 20 iterations (8 subsets). Three subsequent acquisitions were performed with ^99m^Tc background activity concentrations of 0, 6, and 11 kBq/ml (squares, triangles, and circles, respectively). Mean activity concentrations were measured in volumes of interest (VOIs) matching the actual sphere sizes (solid lines), and in VOIs with diameters increased by 20 mm (dotted lines)

The reconstructed ^166^Ho images of the NEMA IQ phantom were analyzed by defining volumes of interest (VOIs) over the spheres that matched the actual sphere sizes. Figure 4 shows the activity recovery for the six spheres in which the expected activity was based on the dose-calibrator measurements (used as ground truth). In this phantom study, activity recovery was highest for the ^166^Ho only images, i.e., addition of ^99m^Tc decreased the activity recovery. When the diameters of the VOIs were increased by 20 mm, however, adding ^99m^Tc increased the apparent ^166^Ho activity.

**Fig. 5:**
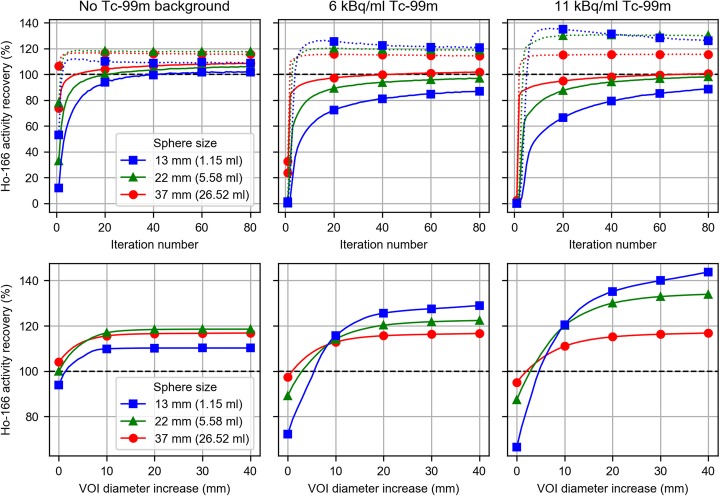
Recovered ^166^Ho activity as a percentage of the known injected activity for ^166^Ho filled spheres in the NEMA image quality phantom for various background concentrations of ^99m^Tc (columns). The top row shows the recovered ^166^Ho activity as a function of the number of UMCS-OSEM iterations (8 subsets per iteration) in VOIs matching actual sphere sizes (solid lines) and VOIs with diameters increased by 20 mm (dotted lines). The bottom row shows the recovered ^166^Ho activity after 20 iterations as a function of increased VOI diameter (i.e., in addition to the actual sphere size). For clarity, results of only 3 out of the 6 NEMA IQ spheres are shown

Figure 5, top row, shows the rate of convergence for 3 different sphere sizes and the dependence on ^99m^Tc background activity concentration. Based on these results, 20 iterations were found to provide an acceptable degree of convergence for all but the smallest spheres. The bottom row in Fig. 5 shows how the increase in VOI diameter influences the recovered activity (after 20 iterations). Increasing the diameter of VOIs beyond 40 mm caused VOIs to start to overlap with neighboring spheres.

**Fig. 6:**
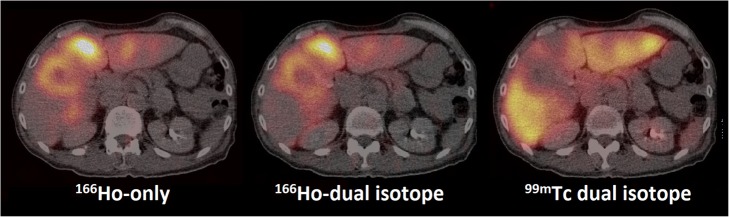
Fused ^166^Ho-only SPECT/CT (left), ^166^Ho-reconstruction SPECT/CT from dual-isotope acquisition (middle), and corresponding ^99m^Tc-stannous phytate reconstruction SPECT/CT from a dual-isotope acquisition (right) in a patient. Visual assessment of the images by two nuclear medicine physicians shows no differences and the ^99m^Tc-stannous phytate SPECT/CT shows a negative correlation (no uptake in tumor tissue, only uptake in healthy liver tissue)

Visual interpretation of the ^166^Ho-only SPECT/CT and the ^166^Ho reconstruction of the dual-isotope SPECT/CT in the patient setting showed no differences, as shown in Fig. 6.

## Discussion

The importance of dosimetry in radioembolization has been emphasized after the negative results of large prospective trials [5–10]. In a post hoc dosimetric analysis of one of these trials, performed in a subset of patients, a clear tumor-absorbed dose—overall survival correlation was found [11, 12]. Patients receiving a sufficient tumor-absorbed dose (≥ 100 Gy) had a significant longer median overall survival and had better objective response rates [11]. Thus, currently a clinical unmet need for improved dosimetry exists which is user/physician friendly. Unfortunately, current available methods or software packages are often very time consuming, incorporate errors by miss registration of different modalities and incorporate additional inter-observer variability/errors by the required manual delineations by the treating physicians.

Based on a previous publication by Lam et al. in 2013, the combination of a pre-treatment simulation SPECT/CT with ^99m^Tc-macroaggregated albumin (^99m^Tc-MAA) and physiological healthy liver tissue delineation with ^99m^Tc-sulfur colloid SPECT/CT seemed feasible [13]. ^99m^Tc-labeled radiocolloids only accumulate in healthy liver tissue via phagocytosis of the colloid particle by Kupffer cells [1, 13]. As Kupffer cells are absent in tumorous tissue, this allows an easy differentiation between tumorous and healthy liver tissue. In the study protocol by Lam et al, healthy liver tissue was delineated on the ^99m^Tc-sulfur colloid SPECT/CT using a 10% threshold of the maximum pixel value, and on the ^99m^Tc-MAA SPECT/CT liver tissue below a 10% threshold was simplified to 0 (i.e., “non-irradiated functional liver tissue”). By subtraction of both images with the according thresholds, 4 different compartments (based on physiological data) could be defined as follows: irradiated tumor, irradiated healthy liver tissue, non-irradiated healthy liver tissue, and tumor necrosis. In the population of 122 patients treated with ^90^Y-loaded microspheres, clinical toxicity data was correlated to the absorbed dose in healthy liver tissue [13].

However, the combination of ^99m^Tc-MAA and ^99m^Tc sulfur colloid is impractical, mainly because of the separate acquisition of the two SPECT/CTs. Furthermore, ^99m^Tc-MAA is known to be a poor predictor of intrahepatic distribution of microspheres [14, 15]. To bring pre-treatment dosimetry to a higher level, a more practical imaging protocol and a more predictive particle are needed. In previous studies, the use of a small amount of ^166^Ho microspheres with an activity of 250 MBq, was determined to be safe and superior to ^99m^Tc-MAA [15–19]. Thus the main aim of this study was to investigate the technical feasibility of a dual-isotope protocol combining ^166^Ho microspheres and ^99m^Tc sulfur colloid.

Based on this work, a quantitative reconstruction framework for dual-isotope scanning of a 250 MBq ^166^Ho scout dose with 50 MBq ^99m^Tc-stannous phytate seems feasible for simultaneous treatment simulation and healthy liver tissue delineation. Both isotopes influence each other’s image quality. The 50 MBq ^99m^Tc seems sufficient for the task of segmentation of the healthy liver, although this work focusses on the ^166^Ho image quality rather than ^99m^Tc (in continuing research the ^166^Ho/^99m^Tc activity ratio is evaluated in a clinical setting, where the quality of the ^99m^Tc reconstruction directly impacts dosimetry). The ^166^Ho photopeak window is hampered by the downscatter from the high energetic (> 1.3 MeV) gamma emissions in the ^166^Ho emission spectrum, bremsstrahlung and the generation of (*K*-shell) lead x-rays in the collimator. Addition of ^99m^Tc activity potentially degrades ^166^Ho image quality further.

Phantom experiments revealed that, in absence of ^99m^Tc, UMCS overestimates the ^166^Ho activity by approximately 10%, when compared with the activity reported by our dose calibrator. This difference can be readily compensated for by applying a cross-calibration factor to the reconstructed images. In the presence of ^99m^Tc however, the accuracy of ^166^Ho activity recovery was reduced, especially when small volumes were considered. Figure 5 indicates that the ^99m^Tc background causes the reconstruction to converge more slowly, most notably in the small spheres. Consequently, for a given number of iterations, the resolution recovery is negatively impacted by ^99m^Tc background. However, when VOIs around the spheres were dilated in order to avoid spill-out due to the limited resolution, the recovered activity was overestimated compared with the no-background case, which may partly be caused by counts from the ^99m^Tc background being attributed to ^166^Ho, due to errors in downscatter correction. Experiments with the ^99m^Tc line source and cylindrical source (Fig. 3) indicate that ^99m^Tc-downscatter simulations contain inconsistencies with respect to the measured projections of up to 10%, which may be due to a combination of causes. One of which may be that material compositions cannot be discriminated from the attenuation correction CT, and a material is assumed by the system (i.e., soft tissue [water] or bone). In case of the line source, which was centered in PMMA scatter material, mass attenuation coefficients of water were applied by UMCS rather than those of PMMA. Other causes may be due to the use of a limited number of pre-simulated PSFs and potential inaccuracies in modeling of the gamma cameras’ energy resolution, peaking imperfections, and dead time effects.

There are several limitations to our reconstruction protocol. In the current protocol, patient breathing was not accounted for. Patient breathing is known to result in an underestimation of actual activity depositions and blur SPECT/CT images [20]. This issue may be resolved by applying breath gating during image acquisition, although this feature is currently not supported on our SPECT/CT imaging devices.

The acquisition time is similar to the widely applied ^99m^Tc-MAA SPECT; however, the post-processing computation time is approximately 3 min per iteration on a regular desktop PC, resulting in a total reconstruction time of 2 h (including 2 reconstructions of 20 iterations and a downscatter simulation). The current implementation of the software lacks multi-processing support, which limits the computational speed, especially given the potential for parallelizable processing. A new version of the software will be developed which will allow for a much greater speedup. In previously conducted prospective studies on ^166^Ho-radioembolization, all patients had both the treatment simulation procedure as well as the actual treatment on the same day (so-called 1-day treatment). Based on our experience with 1-day treatments and current findings, 20 iterations with the current software were clinically acceptable in a daily workflow.

The future potential of this imaging protocol is clear. First, the ^99m^Tc-colloid SPECT has the potential to get (semi-)automated image segmentation, thus avoiding inter-observer differences caused by manual delineation, which is known to result in variations and additional errors in dosimetry [4]. Second, the combined acquisition avoids errors introduced by registration of different modalities.

The results of this study are promising; however, this dual-isotope protocol needs additional research on its applicability in the clinical setting.

## Conclusion

A realistic quantitative reconstruction framework for dual-isotope scanning of ^166^Ho and ^99m^Tc was successfully developed and seems feasible for clinical practice. This dual-isotope protocol may resolve several technical issues in radioembolization dosimetry.

## Data Availability

Not applicable.

## Abbreviations

^166^Ho: Holmium-166
^99m^Tc: Technetium-99m
HEPAR PLUS: Holmium Embolization Particles for Arterial Radiotherapy Plus ^177^Lu-DOTATATE in Salvage NET patients
MAA: Macroaggregated albumin
PSF: Point spread function
SPECT: Single photon emission computed tomography
UMCS: Utrecht Monte Carlo System
VOI: Volume of interest
